# Exploring the Regional Coordination Relationship between Water Utilization and Urbanization Based on Decoupling Analysis: A Case Study of the Beijing–Tianjin–Hebei Region

**DOI:** 10.3390/ijerph19116793

**Published:** 2022-06-01

**Authors:** Ruihua Shen, Lei Yao

**Affiliations:** College of Geography and Environment, Shandong Normal University, Jinan 250014, China; srh2455089583@163.com

**Keywords:** Beijing–Tianjin–Hebei, water footprint, urbanization, decoupling analysis

## Abstract

Understanding the potential association between the urbanization process and regional water shortage/pollution is conducive to promoting the intensive utilization of local water resources. In this study, the water footprint model was used to estimate water utilization status in terms of both water quantity (virtual water footprint (VWF)) and water quality (grey water footprint (GWF)) in the Beijing–Tianjin–Hebei region (China) during 2004–2017. Their potential coordination relationship with the local urbanization process represented by the gross domestic product (GDP), population (POP), and built-up area (BA) was examined using the Tapio decoupling model. The results showed that from 2004 to 2017, (1) VWF in Beijing and Tianjin showed non-significant decreasing trends, with reductions of 1.08 × 10^9^ and 1.56 × 10^9^ m^3^, respectively, while that in Hebei showed a significant increasing trend, with an increase of 5.74 × 10^9^ m^3^. This indicated a gradually increasing water demand in Hebei and decreasing demand in Beijing and Tianjin. In all three regions, the agricultural sector accounted for a relatively high proportion of VWF compared to other sectors. (2) GWF in Beijing, Tianjin, and Hebei all showed declining trends, with reductions of 2.19 × 10^10^, 2.32 × 10^10^, and 1.66 × 10^11^ m^3^, respectively, indicating considerable local water quality improvement. The domestic sector contributed as the main component of GWF in Beijing, while agriculture was the main contributor in Hebei. The major contributor in Tianjin transitioned from the domestic (before 2015) to the agricultural sector. (3) We found good coordination between VWF and GDP in all three regions, as their local economic development was no longer overly dependent on water consumption. However, the expansion of urban built-up area or population would bring about accelerated depletion of water resources. (4) GWF in the three provinces showed good coordination with GDP, POP, and BA in most years, implying that the development of urbanization no longer strongly caused the pollution of water resources. In sum, policymakers should focus on improving agricultural irrigation efficiency and residents’ awareness of water conservation, so as to gradually achieve sustainable water resource management in the BTH region.

## 1. Introduction

Water is an important strategic resource for maintaining ecological balance and promoting economic development [1]. From 2004 to 2017, China’s water consumption and wastewater discharge increased by 4.96 × 10^10^ and 2.18 × 10^10^ m^3^, respectively [2]. Water shortage and pollution have become serious environmental security issues that hinder China’s socioeconomic development [3]. Meanwhile, rapid urbanization development would further bring about huge pressure on regional water security. (1) Urban population explosion, as well as the prosperous development of industry and commerce, exacerbates water consumption [4,5,6]. (2) Excessive pollutant discharge from the industrial, domestic, and agricultural sectors triggers serious water pollution problems and further aggravates the shortage of available water resources [5,7]. The crisis of water resources has become a non-negligible “bottleneck” of regional sustainable socioeconomic development [7,8], which thus attracts the attention of numerous scholars to this hot topic [9].

Previously, official water withdrawal data were commonly used for indicating regional water consumption [10,11]. However, the water content embedded in products and services (virtual water) was often ignored when measuring regional water consumption [12]. However, virtual water accounts for a significant proportion of global water consumption [13], and it is only by considering this portion of water utilization that we can better reflect the regional water consumption. The concept of water footprint (WF) has been introduced as an effective approach for regional water resources research in recent years [14]. WF allows for water resource utilization assessment from the perspectives of both water quantity and quality [10,15]. Thus, the virtual water footprint (VWF) can indicate the quantity of water consumption, including the water content used in the production processes of different regions (e.g., country [16], urban agglomeration [8], basin [17], city [17], etc.), industrial sectors (e.g., agriculture [6,18], import/export trade [19], etc.), or products (e.g., crops [20,21], social infrastructure [22], etc.) [6]. Numerous studies have been carried out to evaluate regional water consumption using the VWF model. Long et al. [23] calculated the VWF of four provinces in Northwest China in 2000, and found that the VWF was a realistic measurement of the consumption and utilization of water resources. Islam et al. [19] calculated both the direct water consumption and virtual water footprints in five Australian cities, and found that the per capita VWF was 8–10 times higher than that of the direct water consumption in all case cities. El-Marsafawy and Mohamed [24] estimated the agricultural VWF in Egypt to assess the general water consumption during the growth phase of crops. In addition, the concept of grey water footprint (GWF) has been proposed as an indicator of water pollution induced by pollutants such as nitrogen and phosphorus [25,26]. GWF evaluates the degree of water pollution by considering the amount of fresh water needed to dilute the water pollutants to meet certain water quality standards [27,28]. It has been found that the regional GWF is higher than the VWF, which indicates that water quality issues would lead to more serious water stress than water quantity consumption [29,30,31]. The neglect of the GWF would thus lead to an underestimation of the regional water assessment. Corredor et al. [32] applied GWF analysis in an artisanal mining region in Colombia, and found that higher water pollution pressure was associated with dumping of suspended solids containing mercury. Chini et al. [33] found significant seasonal variation in water pollution based on estimating the GWF from thermoelectric power plants in the USA. Chen et al. [34] estimated the water quality situation of the irrigated region of Yinchuan (China) using the GWF model and revealed its potential driving factors. Feng et al. [28] quantified the GWF in China from 2003 to 2018, and concluded a continuous deterioration of the surface water quality.

Generally, previous VWF and GWF studies have mostly focused on topics such as regional water environment sustainability [35], driving [17,35], regional transfer, etc. [36,37]. Most found that the WF would be affected by the regional economy, population, and other socioeconomic factors, indicating that the urbanization process has a certain influence on regional water resource utilization [4,38]. Thus, it is necessary to consider the potential constraining effect of urbanization on local water resources [4]. For example, Wang et al. [10] used the WF model to measure water stress in 31 Chinese provinces, and they found a significant spatial correlation between regional water stress and industrial structure. Nayan et al. [39] assessed the impact of urbanization on water resources in the Hyderabad metropolitan region (Pakistan), and found significant overexploitation of groundwater in commercial and high-rise residential regions. Salerno et al. [40] modeled the impact of climate change and urbanization on water quality in Northern Italy, showing that both climate change and urbanization would lead to severe deterioration of surface water quality. Li et al. [4] found that urbanization indicators—such as per capita GDP, and the proportions of secondary and tertiary industry—posed significant impacts on regional water scarcity. Based on the above, a deep understanding of the relationship between urbanization and water resource utilization is crucial to the formulation of regional water resources policies and the promotion of regional sustainable development [4]—especially from the WF perspective.

Addressing these issues, this study selected the Beijing–Tianjin–Hebei (BTH) region in China as the case area, and estimated both the water quantity and quality utilization states of the study region during 2004–2017 using the WF model. Then, gross domestic product (GDP), population (POP), and built-up area (BA) were selected as the urbanization indicators to explore their potential coordination with WF by using the Tapio decoupling analysis. The objectives of this research consisted of the following three main aspects: (1) assessing the water utilization status of the BTH region; (2) exploring the coordination relationship between the regional water utilization status and urbanization; and (3) providing a scientific basis and reference for promoting the synergy regulation and sustainable management of regional water resources.

## 2. Study Area

**Figure 1:**
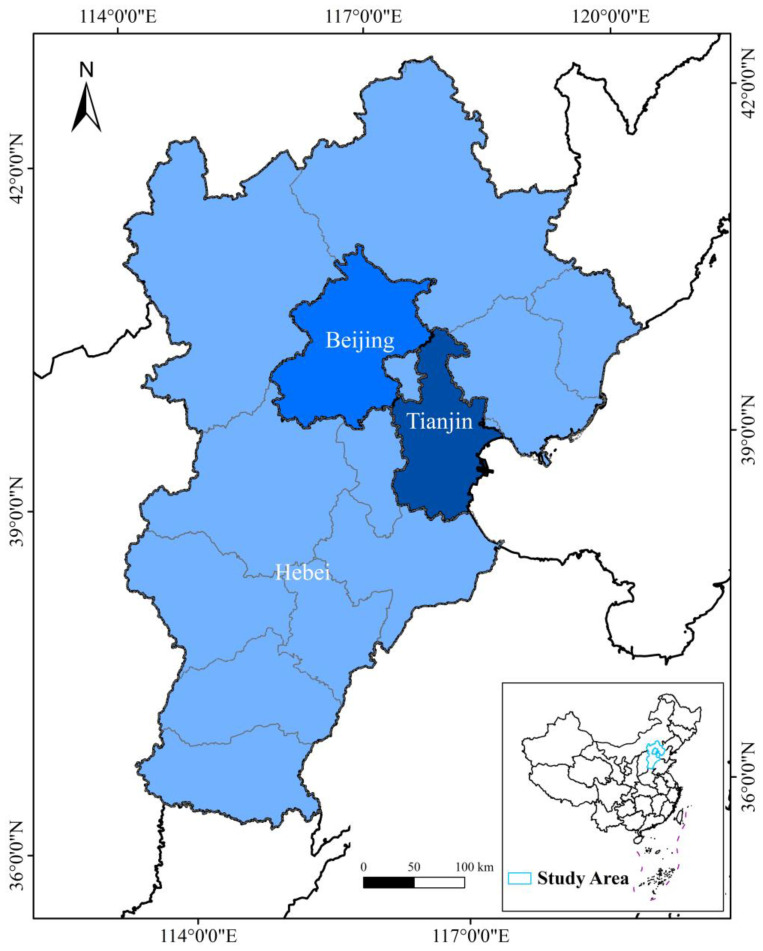
The geographical location of the study area.

The BTH region (located within 36°03′ N to 42°40′ N and 113°27′ E to 119°50′ E, with a total area of ~218,000 km^2^) includes three provincial administrative regions—Beijing, Tianjin, and Hebei [41]—as shown in Figure 1. The three regions share similar climatic conditions and integrated water resource systems [41]. According to the China Water Resources Bulletin in 2017 [42], the total water resources in the BTH region accounted for only 0.6% of China’s water resources, while its wastewater discharge reached 6.8%. In the same year, the region’s population reached 1.10 × 10^6^ (~8% of China), and contributed 8.8% of the whole country’s GDP [43]. As a region with a dense population and a high degree of socioeconomic development, the BTH region is confronting serious water scarcity and pollution issues [44,45,46]. However, there is a significant imbalance in the urbanization levels in Beijing, Tianjin, and Hebei. Beijing and Tianjin are two of the most developed areas (i.e., provincial-level municipalities) in China, while Hebei shows a much lower socioeconomic development level by comparison [13]. In 2017, the urbanization ratio (i.e., the proportion of the urban resident population to the total resident population of the region) in Beijing and Tianjin reached 86.5% and 82.9%, respectively, while in Hebei it was only 55%. Specifically, Hebei (with 11 prefecture-level cities) occupied ~3 times the population size of Beijing, but shared almost the same GDP [47]. With the advancement of regional integration in the BTH region, it is crucial to realize the synergy and equitable utilization of water resources, as well as joint prevention and control of water pollution, in the context of the different urbanization levels of the three areas [18].

## 3. Methodology

**Figure 2:**
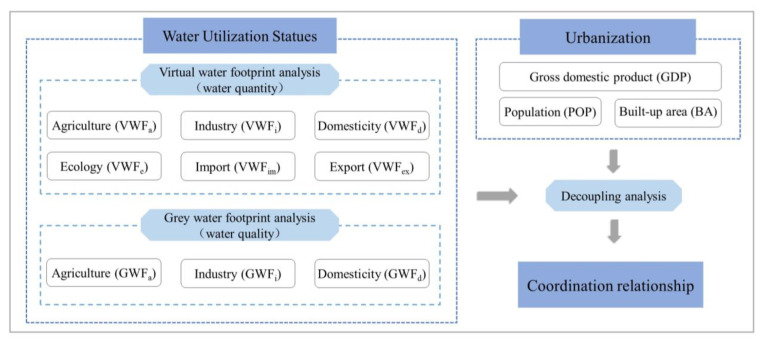
The research framework of this study. VWF_a_: agricultural virtual water footprint, VWF_i_: industrial virtual water footprint, VWF_d_: domestic virtual water footprint, VWF_e_: ecological virtual water footprint, VWF_im_: imported virtual water footprint, VWF_ex_: exported virtual water footprint. GWF_a_: agricultural grey water footprint, GWF_i_: industrial grey water footprint, GWF_d_: domestic grey water footprint.

The general workflow of this study is illustrated in Figure 2. (1) Based on the statistical yearbook data of the BTH region, the year-by-year water resource characteristics were quantified by VWF and GWF analysis. (2) The Tapio decoupling model was used to investigate the coordination relationship between the WF and urbanization indicators.

### 3.1. Data Sources

VWF included six sections (Figure 2): agricultural (GWF_a_), industrial (VWF_i_), domestic (VWF_d_), ecological (VWF_e_), imported (from another region) (VWF_im_), and exported (to another region) (VWF_ex_) [35,48]. (1) Agricultural products were divided into two categories: crops and livestock products. Calculation of VWF_a_ required the annual crop production and livestock breeding data, which were obtained from the local statistical yearbooks [43,49,50] and the *China Regional Economic Statistical Yearbook* [51]. By referring to related studies [35,52,53], 10 types of typical agricultural products in the study region were chosen, and their related virtual water values are listed in Table 1. (2) VWF_i_, VWF_d_, and VWF_e_ are calculations of the industrial, domestic, and ecological water consumption, which were obtained from the *China Statistical Yearbook* [54] and *the China Water Resources Bulletin* [42]. (3) VWF_im_ and VWF_ex_ can be treated as the virtual water volume in the import and export trade in the BTH region (i.e., the trade volume multiplied by the water consumption amount per CNY 10,000 (the Chinese currency) of GDP), and the data on both can be acquired from the *China Statistical Yearbook* [54].

GWF included three sections (Figure 2): agricultural (GWF_a_), industrial (GWF_i_), and domestic (GWF_d_) [34,48]. (1) GWF_a_ required the annual amount of nitrogen fertilizer (N) applied to crops in each region, which were acquired from the *China Environmental Statistical Yearbook* [2] and local statistical yearbooks [43,49,50]. (2) GWF_i_ and GWF_d_ required the data on chemical oxygen demand (COD) and ammonia nitrogen (AN), as well as associated wastewater discharges from the industrial and domestic sectors. These were obtained from the *China Environmental Statistical Yearbook* [2].

In addition, three indicators were chosen to represent the process of urbanization in the study region, including GDP (for economic urbanization), population (for demographic urbanization), and built-up area (for spatial urbanization). All of these indicators were obtained from the *China Statistical Yearbook* [54] and local statistical yearbooks [43,49,50].

Considering the accessibility of all of the abovementioned data in Beijing, Tianjin, and Hebei, the period for obtaining the data involved in this study was set to 2004–2017.

### 3.2. Virtual Water Footprint Analysis

VWF indicates the amount of actual water consumed in the products and services required to sustain the normal life of a regional group under certain physical conditions of living standards, which can be estimated as follows [35,48]:

(1) $$\mathrm{VWF}={\mathrm{VWF}}_{a}+{\mathrm{VWF}}_{i}+{\mathrm{VWF}}_{d}+{\mathrm{VWF}}_{e}+{\mathrm{VWF}}_{\mathrm{im}}-{\mathrm{VWF}}_{\mathrm{ex}}$$

where VWF is the virtual water footprint (m^3^), which indicates the total quantity of water consumed in the study area in a year (m^3^); VWF_a_ is the agricultural virtual water footprint, which expresses the amount of water consumed in agricultural production in the study area in a year (m^3^), and consists of two components—the amount of water used in the production of crop products, and the amount of water used in the production of livestock products—and is calculated by multiplying the virtual water content per unit of agricultural product [16,48]; VWF_i_ is the industrial virtual water footprint (m^3^), which represents the amount of water used for industrial production; VWF_d_ is the domestic virtual water footprint (m^3^), which expresses the amount of water used for domestic use; VWF_e_ is the ecological virtual water footprint (m^3^), which represents the amount of water used for ecological purposes; VWF_im_ is the imported virtual water footprint (m^3^), which indicates the virtual water imported from other regions (m^3^); and VWF_ex_ is the export virtual water footprint (m^3^), which represents the virtual water exported from the study area (m^3^). VWF_im_ and VWF_ex_ are calculated by multiplying the total amount of import and export trade in each region by the water consumption per CNY 10,000 of GDP. Since all other economic indicators are expressed in CNY, the total amounts of import and export trade should be converted from USD (USA dollars) to CNY [35].

### 3.3. Grey Water Footprint Analysis

GWF is defined as the quantity of fresh water required to assimilate pollutant loads to achieve the specific environmental water quality standard [29,34], and it is an effective indicator for quantitatively evaluating the impact of human activities on freshwater systems [55]. It includes the main sources of water pollution in three sectors: agricultural, industrial, and domestic [26,28,34].

#### 3.3.1. Agricultural Grey Water Footprint

GWF_a_ is the amount of fresh water required to carry water pollutants caused by agricultural activities (e.g., livestock manure, livestock house cleaning, fertilizer, and pesticide use) [34]. According to previous studies [25,56], agricultural water pollution is mainly caused by nitrogen (N), including the usage of nitrogen fertilizers and pesticide spraying, etc. Accordingly, the estimation model is as follows:

(2) $${\mathrm{GWF}}_{a}=\frac{\alpha N}{C_{\max}-C_{\mathrm{nat}}}$$

where GWF_a_ is the agricultural grey water footprint (m^3^); α is the rate of nitrogen fertilizer entering the water (%), and the national average nitrogen fertilizer inflow rate of 7% is chosen for calculation, based on previous studies [34,48]; N is the annual amount of nitrogen applied to the crop (kg); C_max_ is the standard concentration of pollutant water quality (kg/m^3^)—with reference to the Environmental Quality Standards for Surface Water of the People’s Republic of China [57], this paper adopts the 3rd level (of a total of 5 levels) as the minimum requirement for wastewater pollution control, with the maximum allowable nitrogen concentration of 1 mg/L—and C_nat_ is the natural concentration of water (kg/m^3^), which is the concentration of water pollution under natural conditions without any anthropogenic interference, and is usually assumed to be 0 [8,26,48].

#### 3.3.2. Industrial Grey Water Footprint

GWF_i_ is the amount of fresh water required to carry the water pollutants caused by industrial production activities [34]. According to the *China Environmental Statistical Yearbook* [2], COD and AN are the main pollutants discharged from industrial wastewater; thus, this study used COD and AN as the key components to calculate GWF_i_ [8,48]—the estimation model is as follows:

(3) $${\mathrm{GWF}}_{i}=\max\left({\mathrm{GWF}}_{i\left(\mathrm{COD}\right)},{\mathrm{GWF}}_{i\left(\mathrm{AN}\right)}\right)$$

(4) $${\mathrm{GWF}}_{i\left(j\right)}=\frac{L_{i\left(j\right)}}{C_{\max}-C_{\mathrm{nat}}}-\mathrm{IWD}$$

where GWF_i_ is the industrial grey water footprint (m^3^); according to Hoekstra’s grey water footprint theory [26], GWF_i_ is determined by the most critical pollutants in industrial wastewater, as the maximum GWF_i(j)_ based on COD or AN (j represents COD and AN); L_i(j)_ denotes the annual discharge of category j pollutants in industrial production (kg); IWD is the total annual discharge of industrial wastewater in the study area (m^3^); C_max_ denotes the pollutant water quality standard concentration, which is 20 mg/L and 1 mg/L for COD and AN, respectively [26]; C_nat_ denotes the concentration of water pollution under natural conditions without any anthropogenic influence—based on the *Water Footprint Assessment Manual* [26], the natural water concentrations of COD and AN are 0 mg/L and 0.015 mg/L, respectively.

#### 3.3.3. Domestic Grey Water Footprint

GWF_d_ is the amount of fresh water required to carry the wastewater pollutants discharged from the domestic activities [34]. Both domestic and industrial wastewater discharge are point source pollution, and are mainly contributed by COD and AN [48], which can be estimated in the same way as for GWF_i_:

(5) $${\mathrm{GWF}}_{d}=\max\left({\mathrm{GWF}}_{d\left(\mathrm{COD}\right)},{\mathrm{GWF}}_{d\left(\mathrm{AN}\right)}\right)$$

(6) $${\mathrm{GWF}}_{d\left(j\right)}=\frac{L_{d\left(j\right)}}{C_{\max}-C_{\mathrm{nat}}}-\mathrm{DWD}$$

where GWF_d_ is the domestic grey water footprint (m^3^); GWF_d(j)_ is GWF_d_ based on COD or AN (j represents COD and AN); L_d(j)_ denotes the annual discharge of category j pollutants in domestic production (kg); DWD is the total annual discharge of domestic wastewater in the study area (m^3^); and C_max_ and C_nat_ denote the water quality standard concentration and natural water concentration of pollutants, respectively, and the values of each parameter are the same as those of GWF_i_ [26].

Accordingly, the total GWF (m^3^) of the study area can be determined by combining the GWF_a_, GWF_i_, and GWF_d_. The calculation formula is as follows:

(7) $$\mathrm{GWF}={\mathrm{GWF}}_{a}+{\mathrm{GWF}}_{i}+{\mathrm{GWF}}_{d}$$

### 3.4. Temporal Analysis of the Water Footprint

The Mann–Kendall (MK) test is a statistical nonparametric rank correlation method that has been widely used for detecting the monotonic time-series trends in environmental elements such as precipitation, runoff, temperature, and water quality [58]. The MK test does not require samples to follow a certain distribution, and is not disturbed by a few outliers; thus, it overcomes the restrictions on the form of series distribution restrictions, and is computationally convenient [59,60]. Therefore, the MK method was chosen to analyze the temporal trend of the WF in this paper. The calculation formula is as follows:

(8) $$\mathrm{Sgn}\left(X_{b}-X_{a}\right)\{\begin{array}{c} =1,\mathrm{if}\;X_{b}-X_{a}>0\\ =0,\mathrm{if}\;X_{b}-X_{a}=0\\ =-1,\mathrm{if}\;X_{b}-X_{a}<0 \end{array}$$

(9) $$S=\overset{n-1}{\underset{a=1}{\sum }}\overset{n}{\underset{b=a+1}{\sum }}\mathrm{Sgn}\left(X_{b}-X_{a}\right)$$

(10) $$\mathrm{Var}\left(S\right)=\frac{n\left(n-1\right)\left(2n+5\right)-\sum_{a=1}^{m}t_{a}\left(t_{a}-1\right)\left(2t_{a}+5\right)}{18}$$

(11) $$Z_{\mathrm{MK}}=\{\begin{array}{c} \frac{S-1}{\sqrt{\mathrm{Var}\left(S\right)}},\mathrm{if}\;S>0\\ 0,\mathrm{if}\;S=0\\ \frac{S+1}{\sqrt{\mathrm{Var}\left(S\right)}},\mathrm{if}\;S<0 \end{array}$$

where Sgn is the sign function; X_a_ and X_b_ denote the WF data in years a and b, respectively; n is the number of data points during 2004–2017; the positive (negative) value of the statistic S indicates the upward (downward) trend of the series [60]; Var(S) is the variance function of the statistic S; m is the number of groups containing equal data within the data series; t_a_ is the number of equal data within a group; and Z_MK_ is the standardized statistic.

In the MK test, the significance level is assumed to be α. When |Z_MK_| ≥ Z_1−α/2_, the original hypothesis of no trend does not hold, and it is considered that there is an upward or downward trend in the series [61]. When Z_MK_ > 0, it indicates an upward trend in the series, and when Z_MK_ < 0, it indicates a downward trend in the series. When |Z_MK_| ≥ 1.28, it means that the confidence level is greater than 90%; when |Z_MK_| ≥ 1.64, it means hat the confidence level is greater than 95%; and when |Z_MK_| ≥ 2.32, it means that the confidence level is greater than 99%.

### 3.5. Decoupling Analysis

Decoupling is a situation in which the total consumption of material energy does not increase with economic growth, but decreases during economic development [48]. The decoupling theory originated in physics [62], but then was introduced to the environmental field to describe the potential relationship between economic development and resource consumption or environmental pressure [63]. In this paper, the Tapio model was chosen to analyze the decoupling state between WF and the urbanization process to evaluate their potential coordination relationship [3].

**Figure 3:**
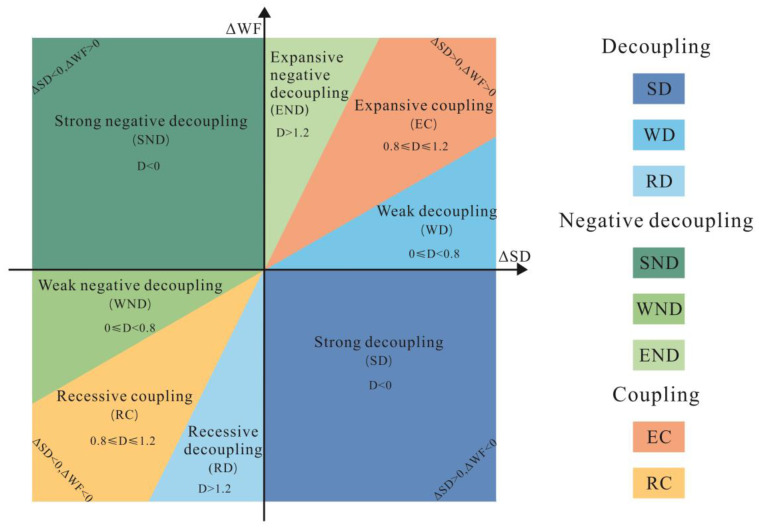
Classification of decoupling states in the Tapio model by referring to Zhang [48] and Kong [35]. The degree of decoupling increases along the positive direction of the ΔSD-axis (change rate of urbanization indicators; horizontal axis) and in the negative direction of the ΔWF-axis (the change rate of the WF; vertical axis).

The Tapio decoupling model is expressed as follows:

(12) $$D=\frac{\Delta \mathrm{WF}}{\Delta \mathrm{SD}}=\frac{{\mathrm{WF}}_{t+1}-{\mathrm{WF}}_{t}}{{\mathrm{WF}}_{t}}/\frac{{\mathrm{SD}}_{t+1}-{\mathrm{SD}}_{t}}{{\mathrm{SD}}_{t}}$$

where D indicates the decoupling index between ΔWF and ΔSD; ΔWF represents the change rate of the WF, including VWF (ΔVWF) and GWF (ΔGWF); ΔSD is the change rate of urbanization indicators, including the GDP (ΔGDP), population (ΔPOP), and built-up area (ΔBA); and t + 1 and t denote year t + 1 and year t, respectively. The Tapio model divides the decoupling states into three categories and eight subcategories according to the three critical decoupling index values of 0, 0.8, and 1.2 [63,64]. Figure 3 and Table 2 show the details of each decoupling state.

## 4. Results

### 4.1. Virtual Water Footprint Analysis

**Figure 4:**
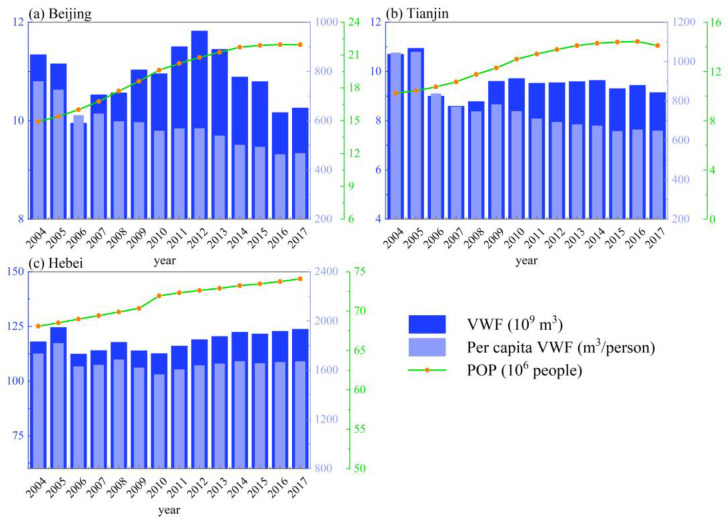
Virtual water footprint (VWF) and per capita VWF of the Beijing–Tianjin–Hebei (BTH) region. POP: population.

The estimated VWF details in Beijing, Tianjin, and Hebei are shown in Figure 4. The results show that the multiyear (2004–2017) average VWF of Beijing, Tianjin, and Hebei was 1.09 × 10^10^, 9.54 × 10^9^, and 1.19 × 10^11^ m^3^, respectively. From the temporal perspective (Table 3), the VWF of Beijing and Tianjin showed non-significant downward trends from 2004 to 2017, decreasing by 9.5% (from 1.13 × 10^10^ to 1.03 × 10^10^ m^3^) and 8.5% (from 1.07 × 10^10^ to 9.75 × 10^9^ m^3^), respectively. In contrast, Hebei showed a significant increasing trend, with a 4.9% increase in VWF from 1.18 × 10^11^ to 1.23 × 10^11^ m^3^. In terms of the per capita VWF, the three provincial regions differed significantly, as Hebei showed the largest average value (1661.0 m^3^/person), while Tianjin and Beijing showed relatively lower values of 763.73 and 577.6 m^3^/person, respectively. From 2004 to 2017, the per capita VWF in all three regions decreased, from 759.7 to 467.7 (Beijing), 1045.3 to 648.7 (Tianjin), and 1733.6 to 1670.7 m^3^/person (Hebei).

**Figure 5:**
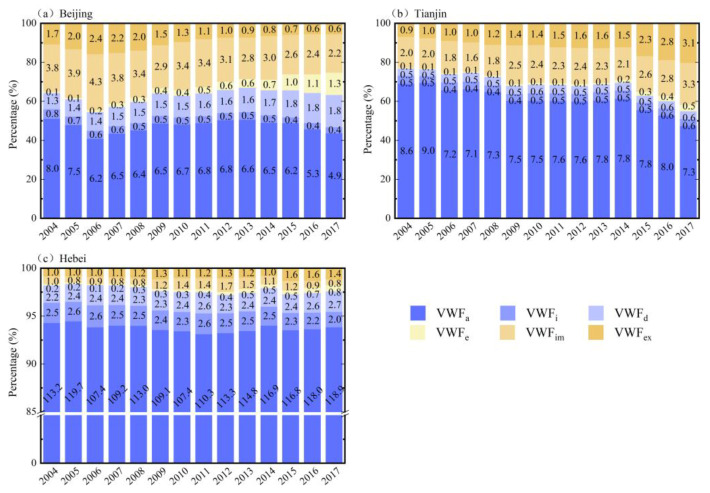
The proportion of VWF (with each component’s amount numbered in black font) in the BTH region.

Figure 5 shows the proportion of each VWF component in the BTH region. It can be seen that there was some similarity in the composition of the VWF of the three case regions. The agricultural sector was the main contributor to VWF, with an average share of 59% (6.48 × 10^9^ m^3^), 81% (7.71 × 10^9^ m^3^), and 94% (1.13 × 10^11^ m^3^) in Beijing, Tianjin, and Hebei, respectively. The domestic sector was the smallest part of the VWF, accounting for less than 5% in all three provinces, but compared with 2004, it had increased by 11% (1.17 × 10^9^ m^3^), 6% (0.47 × 10^9^ m^3^), and 1% (0.62 × 10^9^ m^3^) in Beijing, Tianjin, and Hebei, respectively, in 2017. There were also significant differences between the three regions. In addition to the agricultural sector, the import and domestic sectors were the second- and third-largest contributors to VWF in Beijing, accounting for 27% (2.94 × 10^9^ m^3^) and 15% (1.35 × 10^9^ m^3^), respectively, and VWF_im_ in Beijing had highest proportion among the three areas. Tianjin’s import and export sectors were the second- and third-largest contributors to VWF, accounting for 24% (2.27 × 10^9^ m^3^) and 17% (1.60 × 10^9^ m^3^), respectively, and VWF_ex_ had the highest proportion among the three provinces. Except for agriculture, the contribution of the other sectors in Hebei Province was very small, accounting for no more than 6%.

### 4.2. Grey Water Footprint Analysis

**Figure 6:**
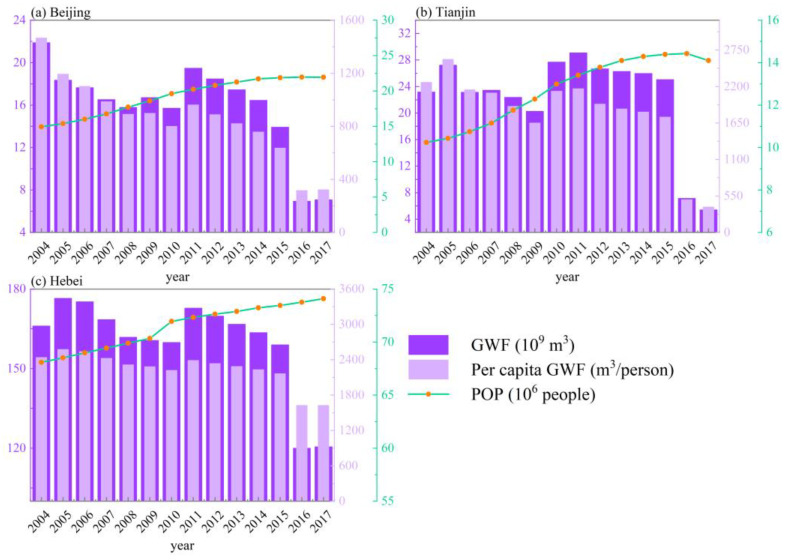
Grey water footprint (GWF) and per capita GWF of the BTH region.

The estimated GWF details in Beijing, Tianjin, and Hebei are shown in Figure 6. The results show that the multiyear (2004–2017) average GWF of Beijing, Tianjin, and Hebei was 1.59, 2.24 × 10^11^, and 1.60 × 10^11^ m^3^, respectively. From the temporal perspective (Table 4), the GWF of Beijing and Hebei showed non-significant (*p* > 0.1) downward trends from 2004 to 2017, decreasing by 67.6% (from 2.19 × 10^9^ to 7.08 × 10^9^ m^3^) and 27.4% (from 1.66 to 1.21 × 10^11^ m^3^), respectively. Meanwhile, Tianjin showed a significant (*p* < 0.05) downward trend, with a 76.6% decrease in GWF from 2.32 × 10^10^ to 5.42 × 10^9^ m^3^. In terms of the per capita GWF, the three regions differed significantly, as Hebei showed the largest average value (2248.0 m^3^/person), while Tianjin and Beijing showed relatively lower values of 1802.3 and 860.7 m^3^/person, respectively. From 2004 to 2017, the per capita GWF in all three regions decreased, from 1466.6 to 322.9 (Beijing), 2665.0 to 384.7 (Tianjin), and 2439.3 to 1627.3 m^3^/person (Hebei).

**Figure 7:**
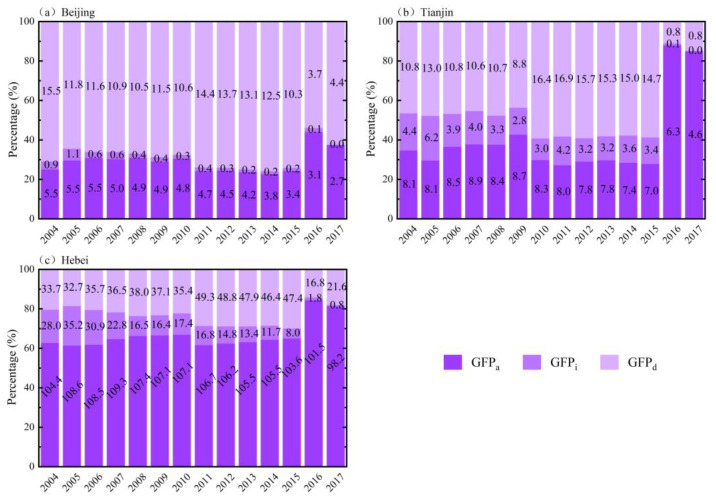
The proportion of GWF (with each component’s amount numbered in black font) in the BTH region.

Figure 7 shows the proportion of each GWF component in the BTH region from 2004 to 2017. Distinct differences in the composition of the GWF were seen in the three case regions. The agricultural sector was the main contributor to GWF in Beijing, with an average share of 68% (1.10 × 10^10^ m^3^), and the change in GWF during the study period was mainly related to GWF_d_. The industrial sector in Beijing contributed the least to GWF, accounting for 2% (0.41 × 10^9^ m^3^) on average. The main contributor to GWF in Tianjin was the domestic sector before 2015, with an average share of 47% (1.14 × 10^10^ m^3^), and the agricultural sector after 2015, with an average share of 40% (7.7 × 10^9^ m^3^). The agricultural sector was the main contributor to GWF in Hebei, accounting for an average of 67% (1.06 × 10^11^ m^3^), which was also the largest among the three provinces. The domestic and industrial sectors contributed relatively little to the GWF of Hebei, with an average proportion of 23% (3.77 × 10^10^ m^3^) and 10% (1.68 × 10^10^ m^3^), respectively, and the GWF_d_ was the smallest among the three provinces.

### 4.3. Decoupling Analysis

#### 4.3.1. Decoupling of the Virtual Water Footprint and Urbanization

**Figure 8:**
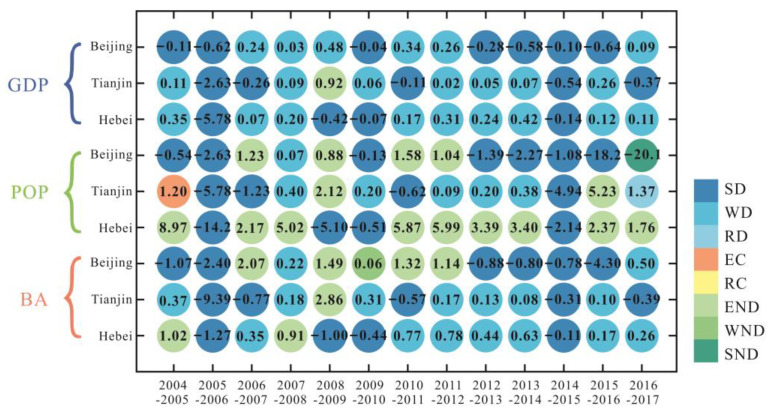
Decoupling state of VWF and GDP (gross domestic product), POP (population), and BA (built-up area) in the BTH region. The value is the decoupling index (D, calculated by Equation (12)). SD: strong decoupling, WD: weak decoupling, RD: recessive decoupling, EC: expansive coupling, RC: recessive decoupling, END: expansive negative decoupling, WND: weak negative decoupling, SND: strong negative decoupling.

Figure 8 represents the decoupling state of VWF with urbanization indicators in the BTH region from 2004 to 2017. In most years, the relationship between VWF and GDP in Beijing, Tianjin, and Hebei was decoupled (SD and WD, all >12 years). For VWF and POP, more than half of the years (8 years) in Beijing were in a decoupling state (SD and WD), with 5 years of negative decoupling (END and SND). In contrast, Tianjin was dominated by the decoupling state (SD, WD, and RD), with only a quarter of the years as coupling (EC) and negative decoupling (END). The decoupling states of VWF and POP in Hebei were dominated by negative decoupling (END, 8 years). In terms of the coordination relationship between VWF and BA, the decoupling results in Beijing indicated that the states were not stable, and there were multiple states of decoupling (SD and WD) and negative decoupling (END and SND). The VWF and BA in Tianjin and Hebei were decoupling (SD and WD) for about 10 years.

#### 4.3.2. Decoupling of the Grey Water Footprint and Urbanization

**Figure 9:**
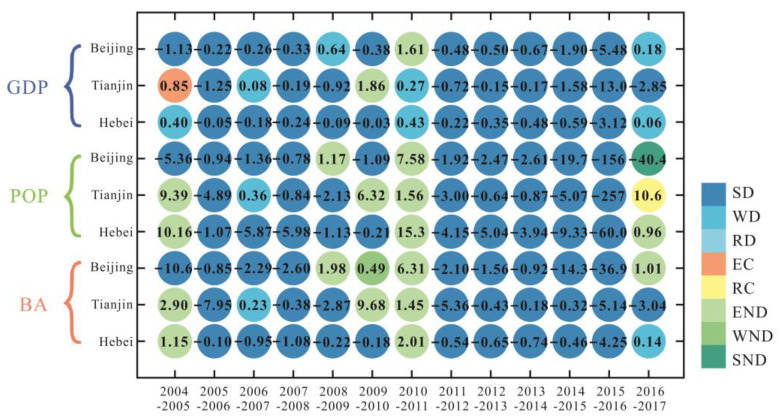
Decoupling state of VWF and GDP, POP, BA in the BTH region. The value is the decoupling index (D, calculated by Equation (12)).

Figure 9 presents the decoupling state of GWF with urbanization indicators in the BTH region from 2004 to 2017. The decoupling results showed that the urbanization indicators and GWF were decoupling (SD and WD) in most years in the BTH region, and there were obvious similarities between the three provinces. In most years, the relationship between GWF and GDP in three provinces was decoupling (SD and WD, all >12 years). The most stable decoupling state was in Hebei province (all 14 years were decoupling states). For GWF and POP, 10 years in Beijing had decoupling states (SD and WD), with 3 years of negative decoupling (END and SND). Tianjin was dominated by a decoupling state (SD and WD), with three years of negative decoupling (END) and one year of coupling (RC). The decoupling states of VWF and POP in Hebei were dominated by decoupling (SD, 8 years), with 3 years of negative decoupling (END). In terms of the coordination relationship between GWF and BA, the decoupling results indicated that the stability of the decoupling state decreased in the following order: Hebei, Tianjin, and Beijing. In addition to the decoupling states, Beijing, Tianjin, and Hebei had four years, three years, and two years of negative decoupling states (END and WND in Beijing; END in Tianjin and Hebei), respectively.

## 5. Discussion

### 5.1. Analysis of Water Resource Utilization in the BTH Region

In this study, we analyzed the water resource consumption and pollution in the BTH region using the VWF model and GWF model, respectively. The VWF results showed that the per capita VWF in Beijing (577.6 m^3^/person) and Tianjin (763.73 m^3^/person) was much lower than the national average (1542.7 m^3^/person) for the same period [65], while that in Hebei was relatively high (1661.0 m^3^/person). This indicates that Hebei was confronted with a relatively high level of water stress. Moreover, from 2004 to 2017, water consumption in Hebei showed a significant increasing trend, and was closely related to population increment (Figure 8), as has also been reported by Kong et al. [35]. The increase in water demand in Hebei should be additionally supplied by the transfer of water from the South–North Water Transfer Project [35].

Hoekstra and Mekonnen [66] confirmed that the agricultural sector contributed the largest share of VWF across the world, especially in China, India, and the USA. Since the BTH region was the second-biggest traditional agricultural area in China [67], the proportion of agricultural water consumption was high in all three provinces, especially in Hebei province (VWF_a_ average >90%)—a largely agricultural province. At the same time, the rough irrigation methods further led to a large amount of water consumption in agricultural production [68]. Water consumption in the ecological sector also increased gradually in the three provinces during the study period (VWF_e_ in Beijing, Tianjin, and Hebei increased by 11%, 6%, and 1%, respectively) due to the rise in the BTH region’s synergistic development through a national strategy in 2014, which strengthened the implementation of various key forestry and ecological projects in the three areas [68]. Both Beijing and Tianjin had a high share of VWF_im_ and VWF_ex_, due to active trade activities and the import and export of water-intensive products. The agricultural sector in Hebei consumed the largest amount of water, while all other sectors accounted for relatively little (< 2%).

With a well-developed service industry and residents’ living standards [68], the domestic sector in Beijing produced the most wastewater discharge. The crude irrigation and aquaculture as well as mechanized livestock farming in Hebei Province also led to a great deal of consumption and pollution of surface water [68]. These all made the problem of water pollution in the study area one of the key threats to water security. Fortunately, the per capita GWF in Beijing, Tianjin, and Hebei (860.7 m^3^/person, 1802.3 m^3^/person, and 2248 m^3^/person, respectively) was significantly lower than the national average level (4542.5 m^3^/person, calculated by Cui et al. [69]), which may be benefits of the relatively advanced wastewater treatment technology and progressively enhanced residential awareness of environmental protection in the BTH region. Moreover, the GWF in all three areas showed decreasing trends. In particular, Tianjin showed a 76.6% reduction in GWF from 2004 to 2017, and a significant improvement in the overall water environment. This was because the synergistic development strategy in Beijing–Tianjin–Hebei had led the three areas to abandon their separate water environment management modes, strengthening the control of the water environment in this region [70]. In addition, the Water Pollution Prevention and Control Action Plan also strengthened the water environment management in the BTH region [71], contributing to a significant reduction in wastewater discharge from the three areas.

### 5.2. Coordinated Relationship between Water Utilization and Urbanization

According to the Tapio decoupling model, it is known that there is a strong link between WF and the urbanization process in the BTH region. When the changes in economic development, population size, and built-up area are stable, the changes in WF will directly affect the decoupling state [48].

There was a good coordination relationship between VWF and GDP in Beijing (all 13 years were decoupling states (SD and WD)) (Figure 8), while the coordination relationship between POP and BA was still weak (all had 5 years of negative decoupling state). With the rapid economic growth, Beijing’s water consumption was growing at a lower rate, or even negatively. However, with the expansion of population size and built-up area, water resource consumption would grow at a faster rate (END) in some years. Even with the decrease in population in Beijing in 2017, water consumption still increased (SND). The coordination relationship between VWF and GDP/BA in Tianjin (Figure 8) was generally good (all had 12 years of decoupling states (SD and WD)); however, the coordination relationship with POP was weaker in comparison, with 2 years of negative decoupling states (END) and 1 year of a coupling state (EC). The relationship between VWF and GDP/BA in Hebei (Figure 8) was in good coordination in most years (with 11 years of decoupling (SD and WD)). Kong et al. [35] also reported a similar decoupling relationship between VWF and GDP in this region (2004–2017), with the WD state being dominant. This indicates that, as the least developed province in the BTH region, Hebei’s economic development is no longer overly dependent on water consumption. However, VWF and POP in Hebei were in a negative decoupling state (END) in most years, and the increase in population would lead to accelerated depletion of water resources. To achieve the decoupling of VWF and POP in Hebei Province, the focus is still on reducing water use for agricultural production, and promoting the progress of agricultural irrigation technology is an important goal [35]. Generally, this emphasizes the coordination relationship between urbanization and water consumption, as the higher the economic level (GDP), the lower the consumption of water resources, whereas the increase in POP and AN would lead to more water consumption, as has also been confirmed by Li et al. [4]. This suggests that urbanization in the BTH region plays a complicated role in influencing regional water resources.

The decoupling state of GWF and the urbanization indicators in the BTH region had strong similarities, and was dominated by decoupling states (SD and WD). Water pollution and urbanization were generally in a good coordination relationship in all three areas (Figure 9). With the rapid development of urbanization, the wastewater discharge was decreasing instead of increasing in most years. This also indicated that the various water pollution control policies enacted for the BTH region had achieved significant results, and guaranteed the healthy development of the urbanization process in the BTH region [70].

### 5.3. Recommendations and Limitations

Based on the main findings of this study, corresponding policy recommendations are proposed to promote the coordination of urbanization and water resource utilization. First, agriculture consumes most of the water resources in the region (Figure 5), and plays a crucial role in achieving the decoupling of VWF and urbanization indicators. Therefore, agricultural water-saving irrigation technology—e.g., agricultural mechanized irrigation—should be vigorously developed to improve water utilization efficiency in parallel with the development of urbanization [35,48]. Second, the increased population will lead to more water consumption (especially in Hebei; Figure 8); thus, the promotion of residents’ awareness of water conservation is essential. Third, we found that the average GWF was much higher than the VWF in all three areas (Figure 4 and Figure 6), which indicates that water pollution has become the main cause of regional water stress compared to the physical water usage in the BTH region [29,30,31]. Moreover, domestic wastewater contributed to the major role of GWF (Figure 7). It is thus necessary to continue to develop wastewater treatment technology for the sustainable development of the region [34], and to raise awareness of water conservation (e.g., stepped price standards to reasonably control domestic water use [48]).

However, this study has some limitations. First, uniform and static parameters (i.e., the virtual water content per unit of agricultural product; Table 1) were adopted to estimate VWF_a_ during the study period. Second, the calculation of GWF only considered the highest level of pollutants, and did not consider the interaction effects of multiple pollutants, which is something worthy of further discussion in the future.

## 6. Conclusions

Water resources are the prerequisite and foundation for regional socioeconomic development. Based on the theory and method of WF, this paper calculated VWF and GWF in the BTH region from 2004 to 2017 to evaluate the situation of water resource utilization. Meanwhile, the Tapio decoupling model was used to analyze the coordination relationship between WF and urbanization. The main conclusions can be summarized as follows:

(1) VWF in Beijing and Tianjin showed a non-significant decreasing trend, with a reduction of 1.08 × 10^9^, 1.56 × 10^9^ m^3^, while Hebei province showed a significant increasing trend, with an increase of 5.74 × 10^9^ m^3^. In all three regions, agriculture accounted for a relatively higher proportion of VWF than other sectors. Improving agricultural irrigation technology should be an important goal in reducing the consumption of water and promoting the decoupling of VWF from the development of urbanization.

(2) GWF in Beijing, Tianjin, and Hebei all showed declined trends, with reductions of 2.19 × 10^10^, 2.32 × 10^10^, and 1.66 × 10^11^ m^3^, respectively. Because of government policy interventions, wastewater discharges in the three provinces were reduced, and the quality of the water environment improved significantly. The domestic sector contributed as the main component of GWF in Beijing, while agriculture was the main contributor in Hebei. The major contributor in Tianjin transitioned from the domestic (before 2015) to the agricultural sector. Future water pollution prevention and control in Beijing should focus on the domestic sector, and in Tianjin and Hebei it should focus on the agricultural sector.

(3) VWF and GDP in the three areas were almost entirely decoupling, and there was a good coordination relationship between VWF and GDP, with economic growth no longer causing an increase in water consumption. In comparison, the coordination relationship between VWF and BA was weaker in Beijing, while that between VWF and POP was weaker in Tianjin and Hebei. In some years, the expansion of the built-up area or the increase in population brought about an accelerated depletion of water resources.

(4) GWF and GDP, POP, and BA were decoupling in most years in the three areas, there was good coordination between GWF and the urbanization process in general, and the development of urbanization has not caused significant pollution of water resources.

## Figures and Tables

**Table 1:** Virtual water per unit of crop products and livestock products (m^3^/kg) [35,52,53].

Products	Virtual Water Content
Grain	1.13
Cotton	4.40
Oil plants	3.97
Fruit	0.82
Vegetables	0.10
Pork	2.21
Beef	12.56
Mutton	5.20
Poultry	3.65
Eggs	3.55
Dairy	1.90

**Table 2:** The meaning of each decoupling state.

Decoupling States	Meaning
Strong decoupling (SD)	Urbanization is accelerating while water consumption (wastewater discharge) is decreasing. At this time, there is the best coordination relationship between urbanization and water utilization.
Weak decoupling (WD)	The increase in the pace of water consumption (wastewater discharge) is smaller than that of urbanization. At this time, there is a better coordination relationship between urbanization and water utilization.
Recessive decoupling (RD)	The decrease in the pace of water consumption (wastewater discharge) is greater than that of urbanization.
Strong negative decoupling (SND)	Urbanization is decreasing while water consumption (wastewater discharge) is increasing.
Weak negative decoupling (WND)	The decrease in the pace of water consumption (wastewater discharge) is smaller than that of urbanization.
Expansive negative decoupling (END)	The increase in the pace of water consumption (wastewater discharge) is greater than that of urbanization.
Expansive coupling (EC)	The increase in the pace of water consumption (wastewater discharge) is approximately equal to that of urbanization.
Recessive coupling (RC)	The decrease in the pace of water consumption (wastewater discharge) is approximately equal to that of urbanization.

**Table 3:** Mann–Kendall test results for virtual water footprint (VWF) during 2004–2017: α indicates the significance level; “Yes” or “No” indicates whether there is a monotonic trend (+: increasing trend with Z_MK_ > 0; −: decreasing trend with Z_MK_ < 0).

Province	Z_MK_	Trend (α = 10%)	Trend (α = 5%)	Trend (α = 1%)
Beijing	−0.547	No	No	No
Tianjin	−0.766	No	No	No
Hebei	2.189	Yes (+)	Yes (+)	No

**Table 4:** Mann–Kendall test results for grey water footprint (GWF) during 2004–2017.

Province	Z_MK_	Trend (α = 10%)	Trend (α = 5%)	Trend (α = 1%)
Beijing	−2.628	Yes (−)	Yes (−)	Yes (−)
Tianjin	−0.985	No	No	No
Hebei	−2.628	Yes (−)	Yes (−)	Yes (−)

## References

[1] Zhang Y.Y., Sun M.Y., Yang R.J., Li X.H., Zhang L., Li M.Y. (2021). Decoupling water environment pressures from economic growth in the Yangtze River Economic Belt, China. Ecol. Indic..

[2] National Bureau of Statistics http://www.stats.gov.cn/ztjc/ztsj/hjtjzl/.

[3] Li Y., Wang Y. (2019). Double decoupling effectiveness of water consumption and wastewater discharge in China’s textile industry based on water footprint theory. PeerJ.

[4] Li W.F., Hai X., Han L.J., Mao J.Q., Tian M.M. (2020). Does urbanization intensify regional water scarcity? Evidence and implications from a megaregion of China. J. Clean. Prod..

[5] Zhao G.L., Liang R.F., Li K.F., Wang Y.M., Pu X.C. (2021). Study on the coupling model of urbanization and water environment with basin as a unit: A study on the Hanjiang Basin in China. Ecol. Indic..

[6] Yerli C., Sahin U. (2021). An assessment of the urban water footprint and blue water scarcity: A case study for Van (Turkey). Brazlian J. Biol..

[7] Yang Y. (2017). Assessment on the Water Risks in Provincial Scale in China. Master’s Thesis.

[8] Zhao D.D., Tang Y., Liu J.G., Tillotson M.R. (2017). Water footprint of Jing-Jin-Ji urban agglomeration in China. J. Clean. Prod..

[9] Zhuo L., Feng B.B., Wu P. (2020). Water footprint study review for understanding and resolving water issues in China. Water.

[10] Wang Y., Zhang Y.H., Sun W.X., Zhu L. (2022). The impact of new urbanization and industrial structural changes on regional water stress based on water footprints. Sustain. Cities Soc..

[11] Pfister S., Koehler A., Hellweg S. (2009). Assessing the environmental impacts of freshwater consumption in LCA. Environ. Sci. Technol..

[12] Makate C., Wang R.C., Tatsvarei S. (2017). Water footprint concept and methodology for warranting sustainability in human-induced water use and governance. Sustain. Water Resour. Manag..

[13] Sun S. (2019). Water footprints in Beijing, Tianjin and Hebei: A perspective from comparisons between urban and rural consumptions in different regions. Sci. Total Environ..

[14] Hoekstra A.Y., Chapagain A.K. (2006). Water footprints of nations: Water use by people as a function of their consumption pattern. Water Resour. Manag..

[15] Shi C.F., Yuan H., Pang Q.H., Zhang Y.Y. (2020). Research on the decoupling of water resources utilization and agricultural economic development in Gansu province from the perspective of water footprint. Int. J. Environ. Res. Public Health.

[16] Hossain I., Imteaz M.A., Khastagir A. (2020). Water footprint: Applying the water footprint assessment method to Australian agriculture. J. Sci. Food Agric..

[17] An M., Fan L.J., Huang J.C., Yang W., Wu H., Wang X., Khanal R. (2021). The gap of water supply-demand and its driving factors: From water footprint view in Huaihe River Basin. PLoS ONE.

[18] Yu D.Y., Ding T.C. (2021). Assessment on the flow and vulnerability of water footprint network of Beijing city, China. J. Clean. Prod..

[19] Islam K.M.N., Kenway S.J., Renouf M.A., Wiedmann T., Lam K.L. (2021). A multi-regional input-output analysis of direct and virtual urban water flows to reduce city water footprints in Australia. Sustain. Cities Soc..

[20] Yang X., Zhuo L., Xie P.X., Huang H.R., Feng B.B., Wu P.T. (2021). Physical versus economic water footprints in crop production: A spatial and temporal analysis for China. Hydrol. Earth Syst. Sci..

[21] Ma W.J., Meng L.H., Wei F.L., Opp C., Yang D.W. (2021). Spatiotemporal variations of agricultural water footprint and socioeconomic matching evaluation from the perspective of ecological function zone. Agric. Water Manag..

[22] Kim Y.W., Hwang Y.W., Jo H.J., Kim J. (2021). Water footprint assessment in expressway infrastructure system. J. Clean. Prod..

[23] Long A.H., Xu Z.M., Zhang Z.Q. (2003). Estimate and analysis of water footprint in northwest China, 2000. J. Glaciol. Geocryol..

[24] El-Marsafawy S.M., Mohamed A.I. (2021). Water footprint of Egyptian crops and its economics. Alex. Eng. J..

[25] Dong H.Y., Zhang L., Geng Y., Li P., Yu C.H. (2021). New insights from grey water footprint assessment: An industrial park level. J. Clean. Prod..

[26] Hoekstra A.Y., Aldaya M.M., Chapagain A.K., Mekonnen M.M. (2011). The Water Footprint Assessment Manual: Setting the Global Standard.

[27] Yapicioglu P., Yesilnacar M.I. (2021). Grey water footprint assessment of groundwater resources in southeastern Turkey: Effect of recharge. Water Sci. Technol. Water Supply.

[28] Feng H.Y., Sun F.Y., Liu Y.Y., Zeng P., Deng L.Z., Che Y. (2021). Mapping multiple water pollutants across China using the grey water footprint. Sci. Total Environ..

[29] Li H., Liang S., Liang Y.H., Li K., Qi J.C., Yang X.H., Feng C.Y., Cai Y.P., Yang Z.F. (2021). Multi-pollutant based grey water footprint of Chinese regions. Resour. Conserv. Recycl..

[30] Wang H.X., Yang Y.X. (2018). Trends and consumption structures of China’s blue and grey water footprint. Water.

[31] Gerbens-Leenes P.W., Hoekstra A.Y., Bosman R. (2018). The blue and grey water footprint of construction materials: Steel, cement and glass. Water Resour. Ind..

[32] Corredor G.J.A., González G.L.V., Velasco Granados M., Gutiérrez L., Pérez E.H. (2021). Use of the gray water footprint as an indicator of contamination caused by artisanal mining in Colombia. Resour. Policy.

[33] Chini C.M., Logan L.H., Stillwell A.S. (2020). Grey water footprints of U.S. thermoelectric power plants from 2010–2016. Adv. Water Resour..

[34] Chen J., Gao Y.Y., Qian H., Jia H., Zhang Q.Y. (2021). Insights into water sustainability from a grey water footprint perspective in an irrigated region of the Yellow River Basin. J. Clean. Prod..

[35] Kong Y., He W.J., Yuan L., Shen J.Q., An M., Degefu D.M., Gao X., Zhang Z.F., Sun F.H., Wan Z.C. (2019). Decoupling analysis of water footprint and economic growth: A case study of Beijing-Tianjin-Hebei region from 2004 to 2017. Intern. J. Environ. Res. Public Health.

[36] Zhang C., Anadon L.D. (2014). A multi-regional input–output analysis of domestic virtual water trade and provincial water footprint in China. Ecol. Econ..

[37] Li H., Li K., Liang Y.H., Yang Z.F. (2021). Uncovering the structure of virtual multi-regional grey water network in China. Resour. Conserv. Recycl..

[38] Fan X., Zhang L., Yuan L., Guo B., Zhang Q., Huang H. (2022). Urbanization and water quality dynamics and their spatial correlation in coastal margins of mainland China. Ecol. Indic..

[39] Nayan N.k., Das A., Mukerji A., Mazumder T., Bera S. (2020). Spatio-temporal dynamics of water resources of Hyderabad Metropolitan Area and its relationship with urbanization. Land Use Policy.

[40] Salerno F., Gaetano V., Gianni T. (2018). Urbanization and climate change impacts on surface water quality: Enhancing the resilience by reducing impervious surfaces. Water Res..

[41] Li B.L., Hu Y.M., Chang Y., Liu M., Wang W.J., Bu R.C., Shi S.X., Qi L. (2021). Analysis of the factors affecting the long-term distribution changes of wetlands in the Jing-Jin-Ji region, China. Ecol. Indic..

[42] National Bureau of Statistics (2017). China Water Resources Bulletin.

[43] Beijing Municipal Bureau Statistics http://tjj.beijing.gov.cn/tjsj_31433/.

[44] Yang Z., Niu G.M. (2019). Analysis on coordinated governance of water pollution in Beijing-Tianjin-Hebei region from basin perspective. Yangtze River.

[45] Wang J., Li Y.R. (2017). Main pollutant discharge and its control in Beijing-Tianjin-Hebei region. Areal Res. Dev..

[46] Ji H., Peng D., Fan C., Zhao K., Gu Y., Liang Y. (2022). Assessing effects of non-point source pollution emission control schemes on Beijing’s sub-center with a water environment model. Urban Clim..

[47] National Bureau of Statistics (2018). China Energy Statistic Yearbook.

[48] Zhang Y., Liu W.X., Cai Y., Khan S.U., Zhao M. (2021). Decoupling analysis of water use and economic development in arid region of China-Based on quantity and quality of water use. Sci. Total Environ..

[49] Tianjin Municipal Bureau of Statistics http://stats.tj.gov.cn/tjsj_52032/tjnj/.

[50] Hebei Municipal Bureau of Statistics http://tjj.hebei.gov.cn/hetj/tjsj/jjnj/.

[51] CNKI National Bureau of Statistics. https://navi.cnki.net/knavi/yearbooks/YZXDR/detail.

[52] Li N., Zhang J.Q., Wang L. (2017). Decoupling and water footprint analysis of the coordinated development between water utilization and the economy in urban agglomeration in the middle reaches of the Yangtze River. China Popul. Resour. Environ..

[53] Sun C., Zhao C.S. (2013). Liangshi Spatial Correlation Pattern Analysis of Water Footprint Intensity Based on ESDA Model at Provincial Scale in China. J. Nat. Resour..

[54] National Bureau of Statistics http://www.stats.gov.cn/tjsj/ndsj/.

[55] Fu T.B., Xu C.X., Yang L.H., Hou S.Y., Xia Q. (2021). Measurement and driving factors of grey water footprint efficiency in Yangtze River Basin. Sci. Total Environ..

[56] Jia X.X., Varbanov P.S., Alwi S.R.W., Yang D., Klemeš J.J. (2021). Cost-based quantitative-qualitative water footprint considering multiple contaminants. Resour. Conserv. Recycl..

[57] Ministry of Ecology and Environment of the People’s Republic of China (2002). GB3838-2002.

[58] Güçlü Y.S. (2020). Improved visualization for trend analysis by comparing with classical Mann-Kendall test and ITA. J. Hydrol..

[59] Yao L., Sun S., Song C.X., Li J., Xu W.T., Xu Y. (2021). Understanding the spatiotemporal pattern of the urban heat island footprint in the context of urbanization, a case study in Beijing, China. Appl. Geogr..

[60] Sang Y.F., Wang Z.G., Liu C.M. (2014). Comparison of the MK test and EMD method for trend identification in hydrological time series. J. Hydrol..

[61] Güçlü Y.S. (2018). Multiple Şen-innovative trend analyses and partial Mann-Kendall test. J. Hydrol..

[62] Tapio P. (2005). Towards a theory of decoupling: Degrees of decoupling in the EU and the case of road traffic in Finland between 1970 and 2001. Transp. Policy.

[63] Zhang X., Geng Y., Shao S., Song X.Q., Fan M.T., Yang L.L., Song J.K. (2020). Decoupling PM2.5 emissions and economic growth in China over 1998-2016: A regional investment perspective. Sci. Total Environ..

[64] Luo H., Li L., Lei Y.L., Wu S.M., Yan D., Fu X.S., Luo X.M., Wu L.K. (2021). Decoupling analysis between economic growth and resources environment in Central Plains Urban Agglomeration. Sci. Total Environ..

[65] Pan Z.W., Xu C.H. (2019). Decoupling analysis of water resources utilization and economic growth in China. J. South China Agric. Univ. Nat. Sci. Ed..

[66] Hoekstra A.Y., Mekonnen M.M. (2012). The water footprint of humanity. Proc. Natl. Acad. Sci. USA.

[67] Wang P.P., Li Y.P., Huang G.H., Wang S.G. (2022). A multivariate statistical input–output model for analyzing water-carbon nexus system from multiple perspectives-Jing-Jin-Ji region. Appl. Energy.

[68] Ning L. (2016). Research on Water Resources Optimal Allocation in Beijing-Tianjin-Hebei Region Based on Water Footprint. Ph.D. Thesis.

[69] Cui S.B., Dong H.J., Wilson J. (2020). Grey water footprint evaluation and driving force analysis of eight economic regions in China. Environ. Sci. Pollut. Res..

[70] Zhuo A.Y., Dong Z.F., Qie H.T., Zhou Q., Peng C. (2021). Study on the joint prevention and control mechanism of water pollution in Beijing-Tianjin-Hebei region. Environ. Prot..

[71] Wang F.F., Wu H.W., Lei K., An L.H. (2021). Integration of the basin water-quality target management technology in Beijing-Tianjin-Hebei region. J. Environ. Eng. Technol..

